# Targeted Disruption of Scytalone Dehydratase Gene Using *Agrobacterium tumefaciens*-Mediated Transformation Leads to Altered Melanin Production in *Ascochyta lentis*

**DOI:** 10.3390/jof6040314

**Published:** 2020-11-26

**Authors:** Johannes W. Debler, Bernadette M. Henares

**Affiliations:** Centre for Crop and Disease Management, School of Molecular and Life Sciences, Curtin University, Bentley, WA 6102, Australia; Johannes.Debler@curtin.edu.au

**Keywords:** *Ascochyta lentis*, scytalone dehydratase, genetic manipulation, DHN-melanin biosynthesis, *Agrobacterium tumefaciens*-mediated transformation

## Abstract

Sustainable crop production is constantly challenged by the rapid evolution of fungal pathogens equipped with an array of host infection strategies and survival mechanisms. One of the devastating fungal pathogens that infect lentil is the ascomycete *Ascochyta lentis* which causes black spot or ascochyta blight (AB) on all above ground parts of the plant. In order to explore the mechanisms involved in the pathogenicity of *A. lentis*, we developed a targeted gene replacement method using *Agrobacterium tumefaciens* mediated transformation (ATMT) to study and characterize gene function. In this study, we investigated the role of scytalone dehydratase (SCD) in the synthesis of 1,8-dihydroxynaphthalene (DHN)-melanin in *Al*Kewell. Two *SCD* genes have been identified in *Al*Kewell, *AlSCD1* and *AlSCD2*. Phylogenetic analysis revealed that *AlSCD1* clustered with the previously characterized fungal SCDs; thus, *AlSCD1* was disrupted using the targeted gene replacement vector, pTAR-hyg-SCD1. The vector was constructed in a single step process using Gibson Assembly, which facilitated an easy and seamless assembly of multiple inserts. The resulting *Al*Kewell *scd1::hyg* transformants appeared light brown/brownish-pink in contrast to the dark brown pycnidia of the WT strain and ectopic transformant, indicating an altered DHN-melanin production. Disruption of *AlSCD1* gene did not result in a change in the virulence profile of *Al*Kewell towards susceptible and resistant lentil varieties. This is the first report of a targeted gene manipulation in *A. lentis* which serves as a foundation for the functional gene characterization to provide a better understanding of molecular mechanisms involved in pathogen diversity and host specificity.

## 1. Introduction

Pathogen survival relies on its ability to withstand adverse conditions, adapt to varying environmental stresses and capability to infect its host. In the case of pigmented fungal pathogens, melanin serves as the first line of defense, which protects the cell from different damaging agents such as UV-radiation, extreme temperatures and oxidative stresses [1]. In some fungal pathogens, melanized appressoria have been reported to contribute to pathogenicity, such as the cases of *Magnaporthe grisea* (anamorph *Pyricularia oryzae*) and *Colletotrichum lagenarium* where albino mutants failed to penetrate the intended host [2,3].

Fungal melanin is synthesized from various precursors and formed by the oxidative polymerization of phenolic or indolic compounds [4]. The most common melanin utilized by fungal species is derived from the polymerization of 1,8-dihydroxynapthalene (DHN) using malonyl-CoA as precursor. The biosynthesis of DHN-melanin follows the polyketide pathway to synthesize the first stable intermediate, 1,3,6,8-tetrahydroxynapthalene (T4HN) through at least three distinct routes facilitated by different non-reducing polyketide synthases (nrPKS) [5,6,7,8,9]. The subsequent sequential steps involve several reduction and dehydration processes to convert T4HN to DHN with the intermediate enzyme, scytalone dehydratase (SCD), catalyzing two dehydration steps: scytalone to 1,4,8-trihydroxynapthalene (T3HN) and vermelone to DHN [10].

The ascomycete pathogen *Ascochyta lentis*, from the class Dothideomycetes, is a major problem to the lentil industry worldwide, as it causes ascochyta blight (AB) or black spot in lentil. This damaging pathogen greatly reduces grain quality and yield by causing lesions on all above ground parts of the plant. It is responsible for losses of about 5–50%, especially in susceptible varieties, due to flower and pod abortion [11,12]. Often the main economic loss is due to the reduced market value as a result of the downgrading attributed to seed discoloration. In order to combat AB in lentils, management strategies such as good cultural practices, fungicide treatment and use of resistant cultivars are being employed [13,14]. However, adaptation of the pathogen that led to a novel pathotype capable of overcoming host resistance has been reported for *A. lentis* [15]. Thus, one of the most sustainable ways to control this disease is through the identification of the mechanisms underlying pathogen virulence and host resistance to provide tools for the development of AB resistant cultivars.

Advances in transformation and gene manipulation technologies have enhanced functional gene characterization in diverse economically important filamentous fungi including *Ascochyta rabiei* [16] and *Ascochyta fabae* [17], both belonging to the same genus as *A. lentis*. In *A. rabiei*, the role of a polyketide synthase was determined to participate in the biosynthesis of 1,8-dihydroxynapthalene (DHN)-melanin pigment, which confers protection towards UV radiation through the targeted replacement of the PKS1 gene using PEG-mediated transformation [16]. On the other hand, deletion of *pksAC* in *A. fabae* through *Agrobacterium tumefaciens*-mediated transformation (ATMT) prevented the production of ascochitine and its derivative ascochital, a secondary metabolite with a possible role in protecting the pathogen against other microbial competitors in the environment [17]. Despite the high relevance of *A. lentis* as a fungal plant pathogen and its devastating impact on crop yield, very little is known about the pathogenicity factors that contribute to disease development in *A. lentis*. The mechanism by which *A. lentis* infects lentil is still poorly understood, in part due to the limited biochemical tools available to study gene function in this pathogen. Recently, an *Agrobacterium tumefaciens* based transformation has been reported for *A. lentis* [18]; however, targeted gene manipulation has not been described for this pathogen.

In this study, we describe the first targeted gene replacement approach in *A. lentis* demonstrated by the specific manipulation of scytalone dehydratase (*SCD*)-like gene in *A. lentis* isolate *Al*Kewell and the subsequent effect on DHN melanogenesis and pathogenicity. Here, we also report the development of a disruption vector using Gibson Assembly (GA), which facilitated simple and seamless assembly of multiple fragments.

## 2. Materials and Methods

### 2.1. Fungal Isolates and Plasmids

*Ascochyta lentis Al*Kewell, obtained from Kewell, Victoria [15,18,19] was grown in half strength potato dextrose agar (PDA, BD Difco, Sparks, MD, USA) supplemented with 30 µg mL^−1^ streptomycin (Astral Scientific, Gymea, Australia) at room temperature under a 12 h fluorescent light regime to induce sporulation. Maintenance and spore preparation of *A. lentis* isolates were performed as described by Davidson et al. (2016) [15]. *Agrobacterium tumefaciens* AGL1 strain was used for the transformation of *Al*Kewell. Plasmids used in this study were transformed in *Escherichia coli* strain OmniMAX 2^TM^ T1 (OM2, Invitrogen, Carlsbad, CA, USA) and grown in LB. All reagents were from Sigma-Aldrich (St. Louis, MO, USA), unless otherwise indicated.

### 2.2. Plant Materials

The lentil accessions used in this study include the susceptible control ILL6002, resistant control ILL7537 together with Australian cultivars PBA Bolt, PBA Hurricane XT and Nipper. Lentil accessions PBA Hurricane XT and Nipper have been previously described [15,18,20] as susceptible and resistant to *Al*Kewell, respectively. Lentil varieties were obtained from Seednet (Horsham, Australia) and Pulse Breeding Australia (Horsham, Australia). Plants were grown as described by Henares et al. (2019) [18].

### 2.3. Sequence and Phylogenetic Analysis

The amino acid sequence of the putative SCD in *Al*Kewell was identified by running InterProScan 5.35–74.0 search on the *Al*Kewell proteome and filtering for annotations containing the scytalone domain (InterPro ID: IPR004235). The phylogenetic tree was generated using the Geneious Tree Builder function of the Geneious Prime software suite version 2020.1.2 (Biomatters Ltd., Auckland, New Zealand) with neighbor-joining algorithm based on multiple sequence alignment of the amino acid sequences. Protein sequences used in the phylogenetic analysis were retrieved from NCBI. Protein IDs are listed in Table S1. Nucleotide sequences of *AlSCD1* and *AlSCD2* were deposited in NCBI and can be accessed using GenBank accession numbers: MT647645 (BankIt2357289 AlSCD1) and MT647646 (BankIt2357292 AlSCD2).

### 2.4. Inhibition Assay Using Tricyclazole

Inhibition of melanin production was monitored using tricyclazole (Sigma Aldrich, St. Louis, MO, USA). Tricyclazole was dissolved in 95% ethanol and dilutions were also made using 95% ethanol. Two mL of ½ PDA containing tricyclazole was dispensed per well of a 12-well plate. Each column of the plate contained a different concentration of tricyclazole which ranged from 0 to 10 µg mL^−1^. Each well was seeded with approximately 50 µL of 1 × 10^4^ spores mL^−1^ of *Al*Kewell and incubated for seven days at room temperature in an incubator with 12/12-h near ultra-violet light/dark cycles regime to induce sporulation.

### 2.5. Construction of Disruption Plasmid for Agrobacterium Transformation

To create a vector for targeted gene replacement via ATMT, we modified pATMT-GpdGFP [18] by removing its GFP expression cassette and inserting two *Kpn*I (NEB, Ipswich, MA, USA) restriction sites between the Pt*rp*C promoter of the hygromycin B cassette and the left border sequence and between the T*trp*C terminator and the right border sequence, respectively. To achieve this, the plasmid backbone was PCR amplified from left to right border using primers JD423 and JD424 (Table 1, Figure S1). The *hph*-cassette was amplified in a separate PCR reaction using primers JD421 and JD422 (Table 1) that carried 24 bp overlapping tails which matched the left and right border sequences of the plasmid backbone, respectively, as well as contained the *Kpn*I restriction sites. The PCR of both reaction fragments was carried out in 10 µL reactions containing 5 µL 2 × Platinum SuperFi PCR Master Mix (ThermoFisher Scientific, Waltham, MA, USA) and 0.5 µM of each primer. Amplification/cycling conditions were: denaturation for 1 min at 98 °C, 35 cycles of 10 s at 98 °C, 10 s at 55 °C and 2 min at 72 °C, final elongation for 5 min at 72 °C. PCR products were separated on a 1% agarose gel (Bioline, Alexandria, NSW, Australia) and then separately purified using the AccuPrep Gel Purification kit (Bioneer Pacific, Kew East, VIC, Australia) according to the manufacturer’s instructions. These fragments were then assembled in a 10 µL reaction mixture containing five µL 2× NEBuilder HiFi DNA Assembly Master Mix (NEB, Ipswich, MA, USA), one µL of the backbone (13 fmol) and 0.5 µL of the *hph*-cassette (22 fmol) fragments and incubated at 50 °C for 60 min to produce pTAR-0-hyg (Figure S1).

### 2.6. Generation of AlKewell AlSCD1 Replacement Mutant

To build the specific *AlSCD1* disruption mutant, 500 bp upstream of the start codon and 500 bp downstream of the stop codon of *AlSCD1* were PCR amplified using primers JD448 and JD449, and JD450 and JD451, respectively (Figure S2). Each primer contains 20 bp of gene specific sequence (Table 1) and 30 bp 5′ extensions. PCR was carried out in a 20 µL reaction containing: 10 µL 2× Platinum SuperFi PCR Master Mix and 0.5 µM of each primer. One µg of pTAR-0-hyg was digested with *Kpn*I*-HF* at 37 °C for 60 min. The restriction fragments and PCR products were separated on a 1.5% agarose gel and then separately purified using the AccuPrep Gel Purification kit according to the manufacturer’s instructions. All four fragments were combined using NEB HiFi DNA assembly Mastermix as described above (Figure S2). The resulting vector, pTAR-hyg-SCD1 was heat shock transformed into *E. coli* OM2 competent cells (Thermo Fisher Scientific, Waltham, MA, USA) for 30 s at 42 °C and plated onto LB plates supplemented with 30 µg·mL^−1^ kanamycin (Astral Scientific, Gymea, NSW, Australia). Putative transformants were screened for correct assembly of the fragments using PCR and verified by Sanger sequencing (Macrogen, Seoul, Korea). The vector was transformed into *A. tumefaciens* AGL1 competent cells by electroporation. Transformation of *A. lentis* strain *Al*Kewell was done using ATMT as described by Henares et al. (2019) [18].

### 2.7. PCR Analysis of Transformants

DNA was extracted from transformed fungal colonies growing on ½ PDA plates containing 50 µg·mL^−1^ hygromycin B (Invitrogen, Carlsbad, CA, USA), 100 µg·mL^−1^ cefotaxime 100 µg·mL^−1^ (Astral Scientific, Gymea, NSW, Australia) and 30 µg·mL^−1^ Streptomycin 30 µg·mL^−1^ (Astral Scientific, Gymea, NSW, Australia) using a quick DNA extraction protocol adapted from Chi et al. (2009) [21] and the detailed protocol was published on protocols.io [22]. PCR reaction to verify gene replacement was carried out using the primer pair JD452 and JD453 listed in Table 1, that amplifies 120 bp upstream of the 5′ flanking region (500 bp 5′ UTR) and 120 bp downstream of the 3′ flanking region (500 bp 3′ UTR), respectively. PCR was carried out using Taq polymerase (Thermo Fisher Scientific, Waltham, MA, USA) as described above.

### 2.8. DNA Isolation and Nanopore Sequencing of AlKewell Isolates

To validate the replacement of *AlSCD1* with *hph* cassette, we carried out MinION^TM^ nanopore sequencing (Oxford Technologies, Oxford, UK) on *Al*Kewell WT, *Al*Kewell *scd1::hyg* (JD202.9 and JD202.22) *AlSCD1*-deficient mutants and one *Al*Kewell ectopic transformant. Isolates were grown in yeast extract dextrose liquid media with constant shaking at 180 rpm for 72 h at 22 °C. Mycelium samples were frozen with liquid nitrogen and ground to a fine powder. DNA was extracted from approximately 500 mg of powdered mycelia using a modified cetyltrimethylammonium bromide (CTAB) method [23,24,25]. DNA concentration and purity were determined using Qubit^®^ 2.0 Fluorometer (Invitrogen, Carlsbad, CA, USA) and NanoDrop Spectrophotometer (Thermo Fischer Scientific, Waltham, MA, USA). DNA integrity was assessed on a 1.5% TBE gel for 60 h using the 5-80 kb protocol on the Pippin Pulse (Sage Science, Beverly, MA, USA).

Libraries were prepared using the SQK-LSK109 ligation sequencing kit (Nanopore Oxford Technologies, Oxford, UK) and the EXP-NBD104 Native Barcoding Expansion 1–12 kit according to the manufacturer’s instructions. Raw fast5 files were basecalled with Guppy 4.2.2, adapters and barcodes were trimmed using qcat 1.1.0 (https://github.com/nanoporetech/qcat) (options: --detect-middle --trim) and sequences were assembled with Canu 2.1.1 [26] (https://github.com/marbl/canu) (options: genomeSize=45m). This draft assembly was polished first once with racon 1.4.16 (https://github.com/isovic/racon) (options: -m 8 -x −6 -g −8 -w 500, as suggested in the medaka manual) and then once with medaka 1.2.0 (https://github.com/nanoporetech/medaka) (options: -m r941_min_high_g360).

The nanopore sequence assemblies and raw reads of *Al*Kewell WT, *Al*Kewell *scd::hyg* JD202.9 and JD202.22, and *Al*Kewell ectopic strains have been deposited in NCBI and can be accessed under BioProject ID 680004 accession PRJNA680004.

### 2.9. Radial Colony Growth

The effect of *AlSCD1* on the growth rate was investigated by monitoring the colony diameter of *Al*Kewell WT, *Al*Kewell *scd::hyg* JD202.9 and JD202.22, and *Al*Kewell ectopic strains on ½ PDA plates in a 13-day time course experiment. A sterile circular (0.5 cm diameter) paper filter disk (Whatman^TM^, Buckinghamshire, UK) was dipped in the spore suspension of approximately 1 × 10^6^ spores·mL^−1^, determined using haemocytometer, and placed in the middle of a ½ PDA plate without hygromycin B. The cultures were allowed to grow in an incubator with 12/12-h near ultra-violet light/dark cycles at room temperature. The diameter of the colony was measured starting from the 6th day until the 13th day with a 2–3 day interval. Measurements were reported as the mean average from three replicates.

### 2.10. Pathogenicity Assay

Pathogenicity of the wild type and transformant strains of *Al*Kewell were assessed as previously described by Henares et al. (2019) [18]. Briefly, spores were prepared from one-week old ½ PDA plates, washed with water, filtered through sterile cotton wool and resuspended in water to a final concentration of 1 × 10^6^ spores mL^−1^. Two-week old plants were spray-inoculated until run-off. Inoculated plants were kept in a chamber with a misting regime of five seconds every two hours for nine days. The temperature was maintained at 18 °C with a 12/12-h photoperiod. Disease symptoms were evaluated for each plant as leaf lesions of the four nodes that was spray inoculated at 14 days post infection (DPI) and scores were reported as % leaf area damage (LAD). Three independent experiments were carried out for each lentil genotype.

### 2.11. Statistical Analysis

Unless otherwise stated, numerical data were analyzed using the statistical program JMP (version 14.3.0, SAS Institute, Cary, NC, USA). To compare means of more than two data sets, analysis of variance (ANOVA) was used followed by Tukey HSD test analysis to distinguish which sets were significantly different from one another. Levels not connected by the same letter are significantly different at *p* < 0.0001.

## 3. Results and Discussion

### 3.1. Identification of Scytalone Dehydratase (AlSCD) Genes in AlKewell

Fungal melanin production follows two different pathways which either involve (1) the generation of 1–3,4-dihydroxyphenylalanine (L-DOPA) through the oxidation of L-tyrosine by tyrosinases or (2) the production of DHN synthesized through the polyketide synthase pathway [4,27,28]. Subsequent polymerization of L-DOPA or DHN leads to melanin that accumulates in the fungal cell wall and other fungal structures exposed to harsh environments [29]. To determine the melanin biosynthetic pathway used by *A. lentis*, isolate *Al*Kewell was grown in the presence of tricyclazole. Tricyclazole is a known inhibitor of the DHN pathway by targeting the reductases (THNRs) that convert the intermediate products T4HN to scytalone and T3HN to vermelone [30,31]. This is often used as a strong indicator that pigmentation is due to DHN rather than from other melanin precursors.

**Figure 1:**
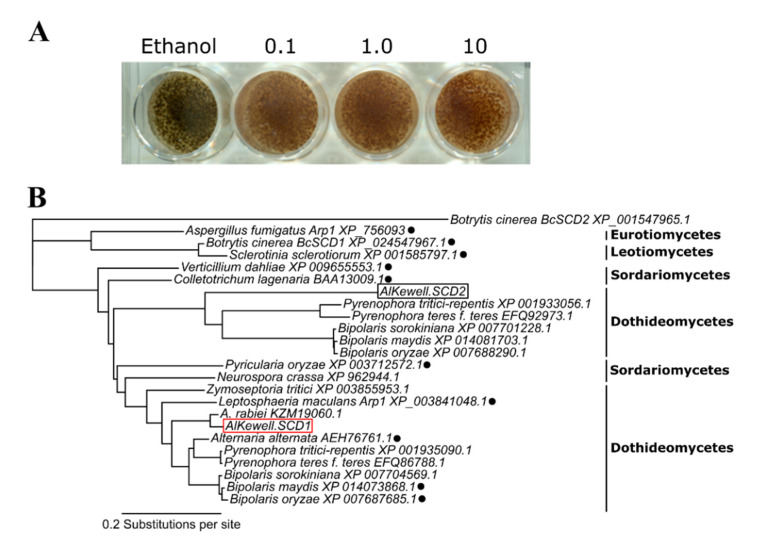
Identification of *scytalone dehydratase* (*AlSCD*) in *Al*Kewell (**A**) Inhibition of melanin production by tricyclazole. Representative photo from three replicates of isolates grown in ½ potato dextrose agar (PDA) supplied with different concentrations of tricyclazole after seven days of incubation. Tricyclazole concentration is expressed as µg mL^−1^ (**B**) Phylogenetic analysis of scytalone dehydratase (SCD) orthologs from different fungal isolates. SCDs that have been previously characterized are indicated by a circle.

*Al*Kewell appeared dark brown when grown in ½ PDA without tricyclazole while light brown/reddish-brown colonies were observed in the presence of the inhibitor (Figure 1A). The reduction of the dark brown pigment was observed at a concentration of as low as 0.1 µg mL^−1^ tricyclazole with no further observable decrease in pigmentation at higher tricyclazole concentrations, which means that 0.1 µg mL^−1^ was sufficient to inhibit the characteristic conidial pigmentation of *Al*Kewell. The appearance of the light brown/reddish-brown pigment in the presence of tricyclazole indicates the accumulation of scytalone, flaviolin and the autooxidation product of T3HN, 2-hydroxyjuglone [7,32] concomitantly, this means that the reduction of the dark brown pigment can be attributed to the inhibition of DHN melanin production. Our finding is in line with the most prevalent DHN biosynthetic pathway used by fungi to synthesize melanin. This contrasts with some fungal plant pathogen species in the class Dothideomycetes that employ the L-DOPA pathway to synthesize melanin such as *Parastagonospora nodorum*, a related pathogen that infects wheat [33] and *Dothistroma septosporum*, a pathogen of pine needle [34].

Scytalone dehydratase is an intermediate enzyme of the DHN pathway that catalyzes two reactions, the dehydration of scytalone to T3HN and vermelone to DHN [10]. We investigated the scytalone dehydratase (*AlSCD*) gene to demonstrate targeted gene disruption in *Al*Kewell and determine the role of melanin in virulence and pathogen survival. In order to identify putative SCD proteins in *Al*Kewell, an InterproScan search for SCD-like domains (ID: IPR004235) was performed on the proteome of *Al*Kewell. We identified two gene homologs in *Al*Kewell, which we referred to as *AlSCD1* and *AlSCD2*. These genes code for 192 amino acids and 164 amino acids, respectively. In *Botrytis cinerea*, two *SCD* genes have been identified in the genome; however, BcSCD1 clustered with the well-studied *A. fumigatus* ARP1 and *Magnaporthe oryzae* RSY1 and has been shown to alter DHN production [7,35,36].

Phylogenetic analysis using the amino acid sequences of SCD orthologs from other DHN melanin-producing fungal species belonging to Eurotiomycetes, Dothideomycetes, Sordariomycetes and Leotiomycetes inferred two major clades (Clades 1 and 2). Analysis revealed that SCDs from *B. cinerea* and *S. sclerotiorum*, members of Leotiomycetes, cluster together and form a distinct clade, alongside with *A. fumigatus* ARP1, belonging to Eurotiomycetes. On the other hand, *Al*Kewell *AlSCD1* and *AlSCD2* (Clade 2) belong to two different subclades where *Al*Kewell *AlSCD1* clusters with SCDs from Sordariomycetes and other Dothideomycetes that have been extensively studied. Specifically, *AlSCD1* is closest to its sister taxa, *A. rabiei* and the previously studied fungal pathogen *Alternaria alternata* Brm1 [37] and *Cochliobolus heterostrophus* (anamorph *Bipolaris maydis*) Sal1 [8] (Figure 1B). As such, *AlSCD1* was chosen for functional characterization.

### 3.2. Disruption of Putative AlSCD1 Gene in AlKewell

Genetic transformation has been previously reported for *A. lentis* [18]; however, targeted gene replacement or knockout has not been described in this pathogen. In order to perform gene replacement in *A. lentis* via ATMT, we developed a vector backbone for gene disruption, pTAR-hyg-0 (Figure S1), through the modification of pATMT-GpdGFP [18]. The vector backbone of pTAR-hyg-0 still carries the hygromycin B phosphotransferase gene cassette (*hph*) as a selectable marker, but with the deletion of the GFP cassette and the insertion of two *Kpn*I sites on the left and right borders to facilitate the addition of target gene sequence for homologous recombination (Figure S1). As such, pTAR-hyg-0 could serve as a versatile vector for genetic manipulation which can be easily treated by digestion with *Kpn*I.

**Figure 2:**
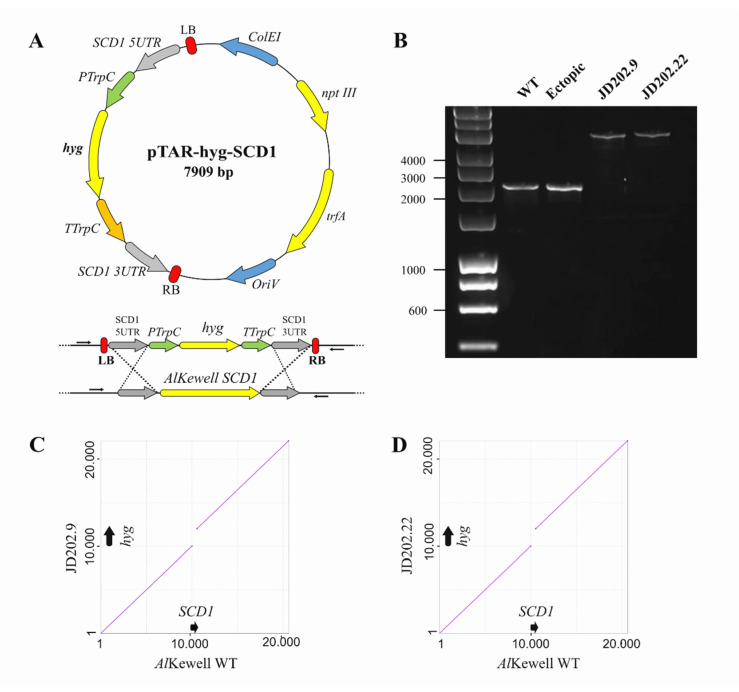
Generation of *Al*Kewell *scd1::hyg*. (**A**) Plasmid map of pTAR-hyg-SCD1, the disruption vector used to target the replacement of *AlSCD1* in *Al*Kewell. (**B**) PCR analysis of *Al*Kewell WT, *Al*Kewell ectopic, *Al*Kewell *scd1::hyg* mutants JD202.9 and JD202.22 using primers JD452 and JD453 indicated as left and right arrows on 2A, respectively. (**C**,**D**) Dot plot comparison of the 20 Kb region around the site of *AlSCD1* integration in *Al*Kewell WT vs *Al*Kewell *scd1::hyg* mutants JD202.9 and JD202.22.

The construction of the specific disruption vector, pTAR-hyg-SCD1, to target *AlSCD1* in *Al*Kewell and replace it with the *hph* cassette (Figure 2A) was formed using Gibson Assembly (GA) of four fragments: 500 bp upstream of *AlSCD1* ORF, 500 bp downstream of *AlSCD1* ORF, *hph* cassette and the vector backbone. The fragments that correspond to the upstream and downstream sequences of *AlSCD1* were amplified from the *Al*Kewell genome using primers with short sequences that overlap with the left and right borders of the vector backbone and P*trpC* and T*trpC* of the hph cassette, respectively (Table 1). The two fragments of the vector backbone were derived from the digestion of pTAR-hyg-0 with *Kpn*I. All four fragments were joined together by an exonuclease, polymerase and DNA ligase in a single isothermal reaction (Figures S1 and S2). Using an enzyme mix in a single reaction eliminated several rounds of PCR amplification and enabled the simple, efficient and seamless assembly of multiple inserts. More importantly, this method has the advantage of producing assemblies with no scar or residual nucleotides [38]. As a result, this strategy would be suitable and could also be employed in the genetic manipulation of other *Ascochyta* species.

Replacement of *AlSCD1* ORF in *Al*Kewell with the functional *hph* gene through homologous recombination was carried out using ATMT. The *Al*Kewell colonies that survived on plates containing hygromycin B were screened using PCR primers that flanked the *AlSCD1* gene up- and downstream of the respective sequences used for homologous recombination (Table 1). The expected size of *AlSCD1* and the flanking regions in the *Al*Kewell wild type (WT) is 1867 bp. This was observed in the *Al*Kewell WT and ectopic transformant, while a much bigger fragment of 3365 bp, which included a fragment of the *Al*Kewell genome (1250 bp) and the *hph* gene cassette (2115 bp) was obtained from *Al*Kewell *scd1*::*hyg* transformants JD202.9 and JD202.22 (Figure 2B). This clearly suggests that a single *hph* gene cassette replaced the *AlSCD1* gene in *Al*Kewell. A total of 24 colonies were screened where two colonies showed positive for disruption of *AlSCD1* while 22 colonies showed random ectopic insertion of the *hph* cassette in the *Al*Kewell genome. We carried out phenotypic analyses on both positive disruption mutants and selected one ectopic mutant which served as negative control for all phenotyping studies in the succeeding experiments.

To verify the replacement of *AlSCD1* with the *hph* cassette, the genomes of the *Al*Kewell WT, two *Al*Kewell *scd1*::*hyg* (JD202.9 & JD202.22) mutants and one *Al*Kewell ectopic transformant were sequenced using the MinION Oxford Nanopore platform. A total of 13 Gbp of data was de novo assembled, resulting in a genome assembly of 42.97 Mbp, 43.21 Mbp, 43.21 Mbp and 42.90 Mbp for *Al*Kewell WT, *Al*Kewell *sch::hyg* (JD 202.9/202.22) and *Al*Kewell ectopic, respectively, with an average read coverage of 21x, 31x, 23x and 23x. Using BLASTn with the *hph* cassette gene sequence as well as the full pTAR-hyg-SCD1 vector sequence against the de novo genome assemblies revealed the integration of the *hph* cassette in the genome of all three transformants. A closer inspection of the T-DNA reads in the genomes of *scd1::hyg* (JD202.9 & JD202.22) mutants showed the complete replacement of the *AlSCD1* gene with a single copy of the *hph* cassette that includes P*trp*C promoter, hygromycin B gene and T*trp*C terminator (Figures S3 and S4). Furthermore, comparative genome analysis of *Al*Kewell WT and *Al*Kewell *scd1::hyg* JD202.9 and JD202.22 genomes confirmed single insertion and that *hph* cassette replaced the *AlSCD1* ORF without altering its reading frame (Figure 2C,D, Figures S3 and S4). Insertion of the *hph* cassette is not found anywhere else in the genome of both *Al*Kewell *scd1*::*hyg* transformants (JD202.9 & JD202.22). On the other hand, mapping of the *hph* cassette to the genome of the ectopic mutant showed that the insertion is off-target and that the *hph* cassette sits in a different contig. Integration of the full disruption vector sequence was not observed for all three mutants. The long reads provided by Nanopore sequencing technology offer the advantage of detecting deletion, replacement or any variation that spans over an entire region. As a result, this technology provides a more accurate view of the genetic variation including identification of copy number. Thus, long read sequencing proves to be an effective alternative method to characterize and assess transformants as compared to Southern Blot method.

Gene targeting strategies based on homologous recombination is a powerful tool to study gene function. The use of ATMT as a vehicle for gene manipulation has been extensively used in many fungal pathogens (reviewed in [39]). Recent advances in gene editing technology has opened a new era for studying functional genomics such as the CRISPR/Cas9 system (clustered regularly interspaced short palindromic repeats/(CRISPR)-associated protein-9 nuclease) by introducing DNA double-strand breaks. Although powerful, this technology still has its limits such as off-target effects and low Cas9 expression which requires optimization for each target for different fungal strains [40]. In this study, we explored the conventional homologous recombination targeting the flanking regions of *AlSCD1* delivered via the ATMT approach to target *AlSCD1* and replace it with a selective marker against hygromycin B, the first report of a targeted gene disruption in *A. lentis*. Gene knockout strategies in limited species of the genus *Ascochyta* have been used to study specific gene function. In *A. rabiei*, a polyketide synthase (*ArPKS1*) was disrupted using a Protoplast-PEG-based transformation in order to determine its role in the production of DHN [16]. On the other hand, the deletion of *PKS* gene (*pksAC*) using ATMT in *Ascochyta fabae* was determined to be responsible for the biosynthesis of the secondary metabolite ascochitine [17]. This demonstration of a gene manipulation strategy in *A. lentis* would enable the examination of genes and putative effectors and their possible role in pathogenicity.

### 3.3. Morphological Characterization of AlSCD1 Disrupted Mutants

**Figure 3:**
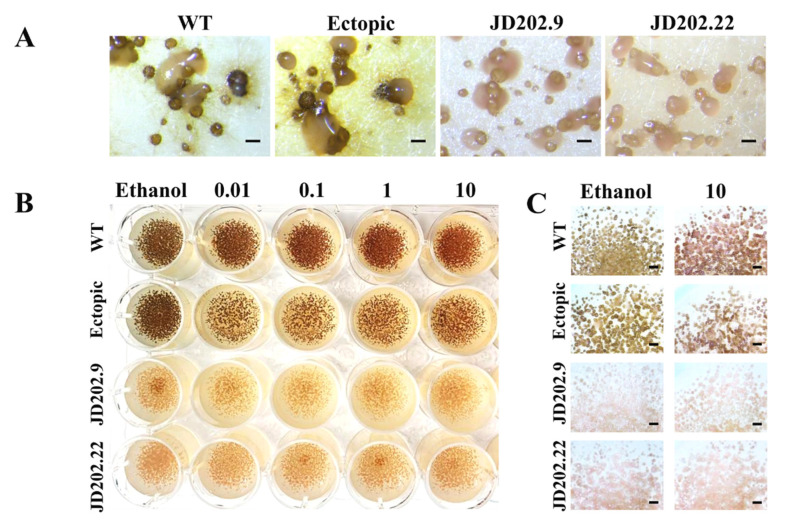
Colony morphology of *Al*Kewell wild type (WT) and transformants grown on ½ PDA for seven days. (**A**) Representative photo from three replicate photos were taken using SMZ-800 stereomicroscope with a DS-Fi1C Digital camera (Nikon Instruments, Melville, NY, USA). Scale bars are 200 µm. (**B**) Inhibition of melanin production in *Al*Kewell WT and transformants using tricyclazole. Representative photo from three replicates of isolates grown in ½ PDA supplemented with different concentrations of tricyclazole grown for seven days. Tricyclazole concentration is expressed as µg mL^−1^. (**C**) Magnification of isolates (from B) grown with (10 µg/mL) and without (ethanol) tricyclazole from three replicate photos taken using SMZ-800 stereomicroscope with a DS-Fi1C Digital camera (Nikon Instruments, Melville, NY, USA). Scale bars are 1mm.

The ability of *Al*Kewell *SCD1-*deficient mutants to form melanin was assessed by growing the transformants that were positive for gene replacement as well as the untransformed *Al*Kewell and ectopic mutant in ½ PDA plates for 14 days. The *Al*Kewell *scd1::hyg* mutants JD202.9 and JD202.22 formed light brown/brownish-pink pycnidia while *Al*Kewell WT formed dark brown pycnidia on PDA plates. Furthermore, insertion of the *hph* gene cassette elsewhere in the *Al*Kewell genome (ectopic) did not result in reduced melanin production but rather the WT characteristic color. This implies that deletion of the *AlSCD1* gene is enough to impair DHN production (Figure 3A and Figure S5).

Involvement of *AlSCD1* in the DHN pathway was further investigated through the inhibition of the melanin intermediates using tricyclazole. Inhibition of DHN melanin production evidenced by the reduction of dark brown pigment was observed in *Al*Kewell WT and ectopic transformant at 0.01 µg mL^−1^ tricyclazole and reduction of pigmentation by the inhibitor was observed to respond in a concentration dependent manner. In contrast, both *Al*Kewell *scd1*::*hyg* mutants JD202.9 and JD202.22 appeared very light brown in the absence of tricyclazole and remained insensitive to tricyclazole even at the highest inhibitor concentration of 10 µg mL^−1^ as shown in Figure 3B (and Figure S6). This data further indicates that in *Al*Kewell, *AlSCD1* participates in the DHN biosynthesis and that disruption of this gene results to altered DHN melanin production. It has been shown in other fungal organisms that disruption of the *SCD1* gene results in the accumulation of intermediate products, possibly scytalone and/or vermelone, hence the failure to produce DHN and the subsequent DHN polymerization. Accumulation of scytalone has been previously reported in albino *SCD1* mutants of *Bipolaris oryzae* [41] and the salmon-colored *Sal1* mutants of *Cochliobolus heterostrophus* (anamorph. *Bipolaris maydis*) [8,42]. Our results are consistent with several fungal species such as *S. sclerotiorum* [43], *B. oryzae* [41], *C. lagenarium* [44], and *M. grisea* [45] where scytalone dehydratase acts as an intermediate enzyme in the DHN-melanin biosynthetic pathway.

**Figure 4:**
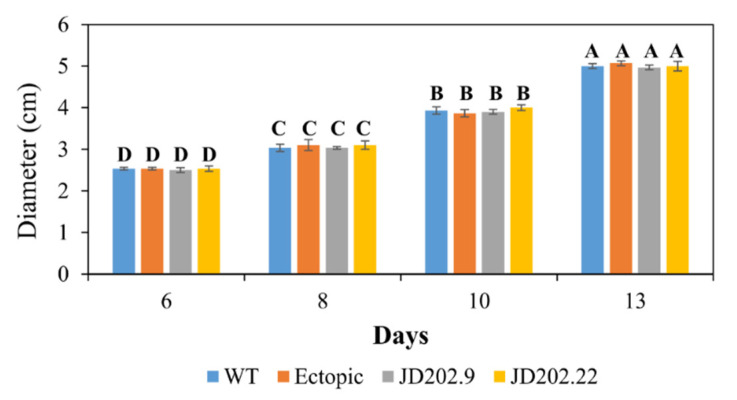
Colony growth of *Al*Kewell and transformants on ½ PDA plates. Each bar is the mean of three replicates and error bars are expressed as standard error of the mean. Bars not connected by the same letter are significantly different (Tukey HSD test, *p* < 0.001).

Fungal melanin has been associated with several developmental and pathogenic characteristics. To determine the effect of deleting the *AlSCD1* gene in the developmental process of *A. lentis*, the radial colony growth of *Al*Kewell WT and the transformants on ½ PDA plates were monitored in the course of 13 days. The diameter of the colony was measured every 2–3 days starting on day six. Colony growth increased steadily from approximately 2.5 cm for all three strains from day six and doubled in diameter after 13 days. No significant difference in diameter was observed between *Al*Kewell *scd1::hyg* JD202.9, JD202.22 transformants and the ectopic transformant (Tukey HSD test, *p* = 0.7367 for isolates; *p* < 0.0001 for days) with the growth rate of the transformants comparable to the wild type (Figure 4). This clearly suggests that neither *AlSCD1* nor melanin alter growth and development of *Al*Kewell in vitro on ½ PDA plates. A similar observation was made in the albino mutants of *Grosmannia clavigera* with *pks* and *scd* gene disruptions [46], and *A. rabiei* Ar20 with a disrupted *pksAC* gene [16], which all had growth rates comparable to their respective WT. In contrast, alteration of melanin production in plant fungal pathogens, *S*. *sclerotiorum* [43] and *A. rabiei* AR628 [16], and the human pathogen *Lomentospora prolificans* [47] showed a significant reduction in the growth rate of the albino mutants.

### 3.4. Pathogenicity of AlSCD1-Targeted Mutants

**Figure 5:**
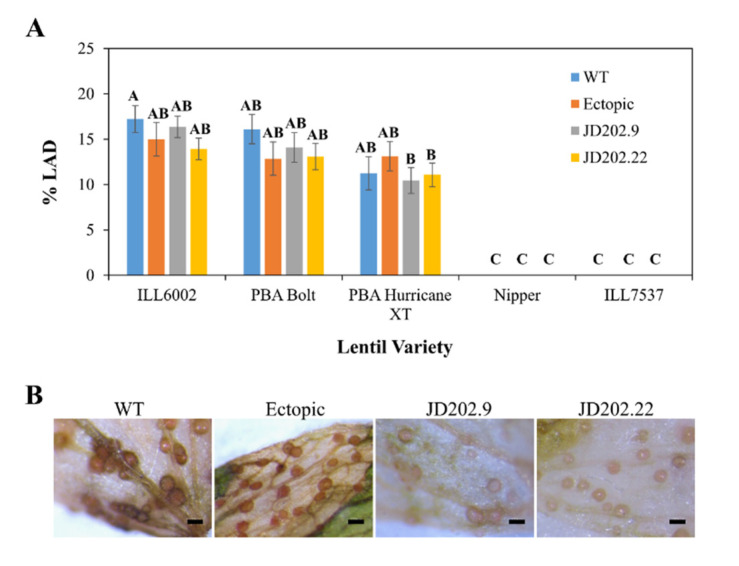
Pathogenicity test of *Al*Kewell and transformants on different lentil varieties. (**A**) Leaf lesions of the four nodes that were spray inoculated (% leaf area damage) are reported as mean values from nine plants of each variety and error bars reported as standard error of the mean. Bars not connected by same letter are significantly different (Tukey HSD test, *p* < 0.0001). (**B**) Representative photo from three replicates of pycnidia on susceptible lentil accession ILL6002 at 14 DPI. Photos were taken using SMZ-800 stereomicroscope with a DS-Fi1C Digital camera (Nikon Instruments, Melville, NY, USA). Scale bars are 200 um.

Melanin production has been reported to aid pathogenicity in *C. lagenarium* and *M. grisea* [2,3]. The ability of the *AlSCD1* disrupted mutant to cause disease was assessed on five lentil varieties using whole plant infection assay. No significant difference in the disease score was observed between *Al*Kewell WT, *Al*Kewell *scd1::hyg* JD202.9 and JD202.22 mutants, and the ectopic transformant (Tukey HSD test, *p* = 0.3734 for isolates; *p* < 0.0001 for lentil varieties). Similar to *Al*Kewell WT, the transformants were able to infect the susceptible lentil control variety ILL6002 and the varieties PBA Bolt, PBA Hurricane XT while the resistant lentil control variety ILL7537 as well as Nipper were not able to elicit a response from the transformants similar to the *Al*Kewell WT (Figure 5A). Further investigation of the infected leaves showed that the pycnidia of *Al*Kewell *scd1::hyg* JD202.9 and JD202.22 mutants had an albino phenotype, while the *Al*Kewell WT and ectopic transformant appeared dark brown (Figure 5B and Figure S7). These results indicate that *AlSCD1* is not essential in the colonization of the susceptible lentil hosts and does not act as a pathogenicity factor in *A. lentis*.

Disruption of DHN-melanin biosynthesis as a result of *AlSCD1* gene replacement did not affect the pathogenicity of *Al*Kewell and this is consistent with the observations in other fungal species that also belong to the class Dothiodeomycetes. A similar observation was also reported for *A. alternata*, where *ALM*, *BRM1, BRM2* mutants had similar necrotic activity on the susceptible pear leaves as the wild type strain, demonstrating that melanin is not essential for virulence [37]. In addition, disruption of the melanin biosynthetic pathway, with regard to the disruption of *ArPKS1*, in *A. rabiei* did not alter its virulence towards chickpea [16]. Both these pathogens synthesize DHN and deposit melanin pigment in both pycnidia and pseudothecia fruiting bodies [16,48] but naturally produce unmelanized appressoria [49]. In the case of *A. lentis*, we did not observe melanin deposition in spores, hyphae or appressoria [50], consistent with its close relative *A. rabiei*. Thus, it can be inferred that penetration of host cells is not mediated by melanized appressoria. In contrast, *Magnaporthe* and *Colletotrichum* species, members of Sordariomycetes, have demonstrated that melanized appressoria are essential for penetration of host cells [2,3,51]. Melanin mediates the build-up of turgor pressure in the appressorium which serves as the driving force for mechanical penetration of the plant cuticle and cell wall [1,52]. Our findings support the observation made in several fungal species that melanin may also play a different role not involved in pathogenesis, but rather it could confer the cell with protection, enhancing its survival under adverse conditions [8,16,43].

## 4. Conclusions

Genetic manipulation plays a major role in studying gene function in vivo. Targeted gene knockout is usually accomplished by using protoplast-polyethylene glycol (PEG)-based transformation or *Agrobacterium tumefaciens-*mediated transformation as the major methods employed in fungal plant pathogens. Gene knockout strategies in limited species of the genus *Ascochyta* have been used to study specific gene function. In the present study, we have demonstrated that *Al*Kewell is amenable to targeted gene manipulation using ATMT, the first report of targeted gene disruption in *A. lentis*. The establishment of a targeted gene replacement strategy in isolate *Al*Kewell provides the foundation to study effector functions and elucidate the genetic mechanism of *A*. *lentis* pathogenesis.

## Figures and Tables

**Table 1:** Primer sequences used in this study.

Primer Name	Sequence (5′ → 3′)	Description
JD421	***ATTGCGGACGTTTTTAATGTACTG*GGTACCC**AGAAGATGATATTGAAGGA	Forward primer *hph* cassette
JD422	***CCCAAATCAAGTTTTTTGGGGTCG*GGTACC**CTCTAAACAAGTGTACCTGT	Reverse primer *hph* cassette
JD423	CGACCCCAAAAAACTTGATTTGGG	Forward primer LB
JD424	CAGTACATTAAAAACGTCCGCAAT	Reverse Primer RB
JD448	***CATTGCGGACGTTTTTAATGTACTGGGTAC***AGGACGAACCATGTTTGCAT	Forward primer *AlSCD1* 5′ UTR
JD449	***AGTGCTCCTTCAATATCATCTTCTGGGTAC***GATGAGTTTGGTGTGTGTGA	Reverse primer *AlSCD1* 5′ UTR
JD450	***AATGCACAGGTACACTTGTTTAGAGGGTAC***GGTGGAGTCTGAGGATATTG	Forward primer *AlSCD1* 3′ UTR
JD451	***ACCCAAATCAAGTTTTTTGGGGTCGGGTAC***GTGAGCCAGCTGCGAGGGGC	Reverse primer *AlSCD1* 3′ UTR
JD452	AGCGCAATGACAAATAGTGC	Forward primer 120 bp upstream5′ flanking region
JD453	CTGTGCCTCGCGCCGCTGCA	Forward primer 120 bp downstream 3′ flanking region

Bold & italic are 5′extensions used for Gibson assembly; underlined are the introduced restriction sites.

## Supplementary Materials

The following are available online at https://www.mdpi.com/2309-608X/6/4/314/s1, Figure S1. Construction of disruption plasmid, pTAR-Hyg-0 from pATMT-GpdGFP, Figure S2. Construction of plasmid used in the targeted gene replacement of AlSCD1 in *Al*Kewell, Figure S3. Integration and coverage of hph-cassette in *Al*Kewell JD202.9, Figure S4. Integration and coverage of hph-cassette in *Al*Kewell JD202.22, Figure S5. Colony morphology of *Al*Kewell WT and transformants, Figure S6. Tricyclazole inhibition of *Al*Kewell WT and transformants, Figure S7. Appearance of *Al*Kewell WT and transformants on the surface of ILL6002 14 days post infection, Table S1. List of SCD orthologs from other fungal organisms, Table S2. Summary of genome assembly statistics for Nanopore sequencing of *Al*Kewell WT and transformants.
