# Supplementary material for: Targeted Disruption of Scytalone Dehydratase Gene Using Agrobacterium tumefaciens-Mediated Transformation Leads to Altered Melanin Production in Ascochyta lentis
Source: J Fungi (Basel). 2020 Nov 26;6(4):314. doi: 10.3390/jof6040314 (PMC7712762; doi:10.3390/jof6040314)
Supplement: Supplementary file 1 [file jof-06-00314-s001.zip › Table S1.docx]

Supplementary Table 1.

| **Organism** | **NCBI accession** | **Protein ID** | **AA** | **Class** |
| --- | --- | --- | --- | --- |
| *Aspergillus fumigatus* | AFUA_2G17580 | XP_756093.1 | 168 | Eurotiomycetes |
| *Magnaporthe oryzae*  *(Pyricularia oryzae)* | MGG_05059 | XP_003712572 | 172 | Sordariomycetes |
| *Neurospora crassa* | NCU07823 | XP_962944 | 174 | Sordariomycetes |
| *Verticillium dahliae* | VDAG_03393 | XP_009655553 | 198 | Sordariomycetes |
| *Colletotrichum lagenarium* | SCD1 | [BAA13009.1](https://www.ncbi.nlm.nih.gov/protein/1395160) | 188 | Sordariomycetes |
| *Alternaria alternata* | HM486908 | AEH76761.1 | 185 | Dothidiomycetes |
| *Bipolaris oryzae (2)* | (1)COCMIDRAFT_94596  (2)COCMIDRAFT_37042 | XP_007687685.1  XP_007688290.1 | 186  162 | Dothidiomycetes |
| *Pyrenophora teres f. teres (2)* |  | EFQ92973  EFQ86788 | 164  187 | Dothidiomycetes |
| *Pyrenophora tritici-repentis (2)* | (1)PTRG_04757  (2)PTRG_02723 | XP_001935090.1  XP_001933056.1 | 187  164 | Dothidiomycetes |
| *Ascochyta rabiei* | ST47_g9804 | KZM19060 | 192 | Dothidiomycetes |
| *Leptosphaeria maculans* | LEMA_P089780.1 | XP_003841048.1 | 187 | Dothidiomycetes |
| *Parastagonospora nodorum* | SNOG 11546 | XP_001801786.1 | 114 | Dothidiomycetes |
| *Zymoseptoria tritici (Mycospaerella graminicola)* | MgSCY1 | XP_003855953.1 | 155 | Dothidiomycetes |
| *Bipolaris sorikiniana* | (1)COCSADRAFT_144875  (2)COCSADRAFT_101310 | XP_007701228.1  XP_007704569.1 | 162,  185 | Dothidiomycetes |
| *Bipolaris maydis*  *(Cochliobolus heterostrophus)* | (1)COCC4DRAFT_164207  (2)COCC4DRAFT_206858 | XP_014081703.1  (ABK63478.1) XP_014073868.1/  BAJ83963 | 162  185 | Dothidiomycetes |
| *Botrytis cinerea (2)* | Bcscd1  Bcin03g08110  Bcscd2  XM_001547915 | XP_024547967.1  [XP_001547965.1](https://www.ncbi.nlm.nih.gov/protein/154295055) | 167  172 | Leotiomycetes |
| *Sclerotinia sclerotiorum* | SS1G_13314 | [XP_001585797.1](https://www.ncbi.nlm.nih.gov/protein/156034757) | 167 | Leotiomycetes |
