# Supplementary material for: Targeted Disruption of Scytalone Dehydratase Gene Using Agrobacterium tumefaciens-Mediated Transformation Leads to Altered Melanin Production in Ascochyta lentis
Source: J Fungi (Basel). 2020 Nov 26;6(4):314. doi: 10.3390/jof6040314 (PMC7712762; doi:10.3390/jof6040314)
Supplement: Supplementary file 1 [file jof-06-00314-s001.zip › Table S2.docx]

Supplementary 2 Table. Summary of genome assembly statistics for Nanopore sequencing of *Al*Kewell WT and transformants

|  | *Al*Kewell WT | A*l*Kewell *scd1::hyg*  JD202.9 | *Al*Kewell *scd1::hyg*  JD202.22 | *Al*Kewell ectopic |
| --- | --- | --- | --- | --- |
| Total bps sequenced | 1,349,766,563 | 1,997,593,686 | 1,429,983,014 | 1,432,872,288 |
| Genome Assembly size Mb | 45.36 | 45.90 | 45.10 | 44.50 |
| Coverage | 29 | 43 | 31 | 32 |
| Number of contigs > 500 bp | 372 | 303 | 281 | 228 |
| Largest contig | 1,423,139 | 1,972,268 | 1,636,978 | 1,918,187 |
| N50 (contigs) | 459,377 | 943,129 | 529,969 | 653,222 |
| L50 (contigs) | 32 | 16 | 31 | 22 |
