# Supplementary figures and images for: Targeted Disruption of Scytalone Dehydratase Gene Using Agrobacterium tumefaciens-Mediated Transformation Leads to Altered Melanin Production in Ascochyta lentis
Source: J Fungi (Basel). 2020 Nov 26;6(4):314. doi: 10.3390/jof6040314 (PMC7712762; doi:10.3390/jof6040314)

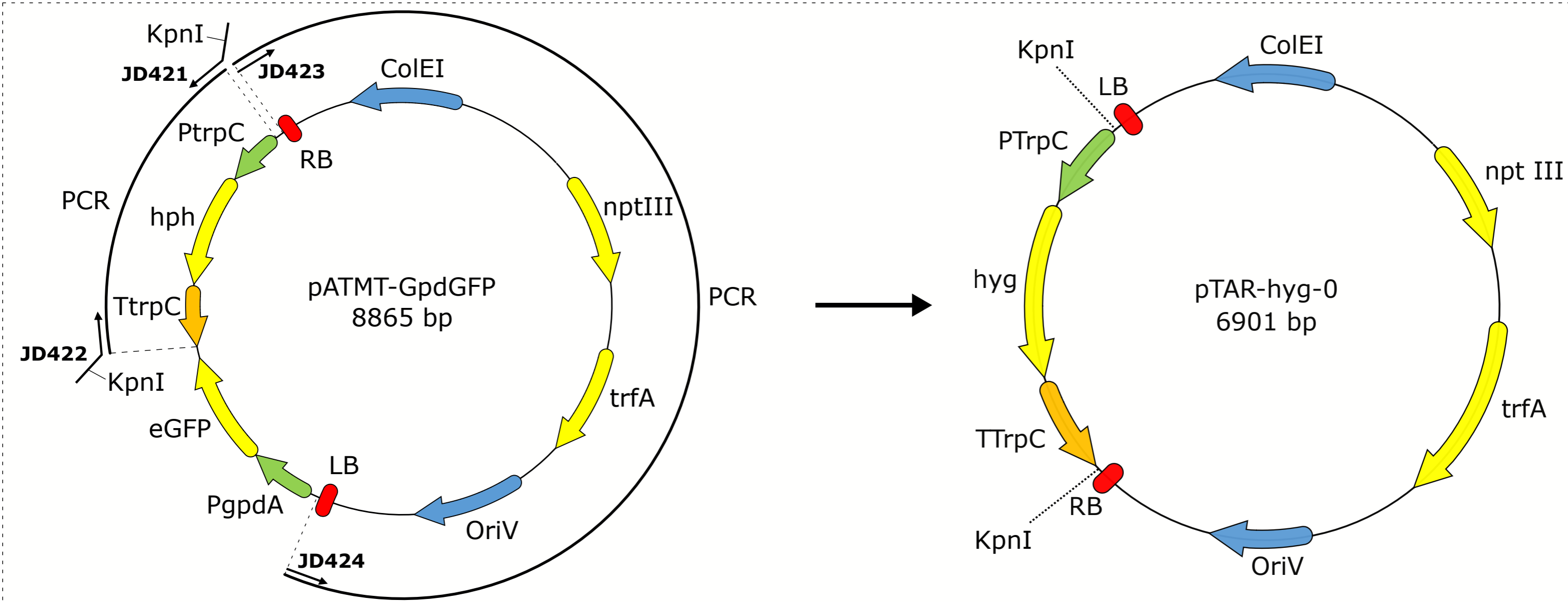

Supplement: Supplementary file 1 [file jof-06-00314-s001.zip › Figure S1.pdf]

A

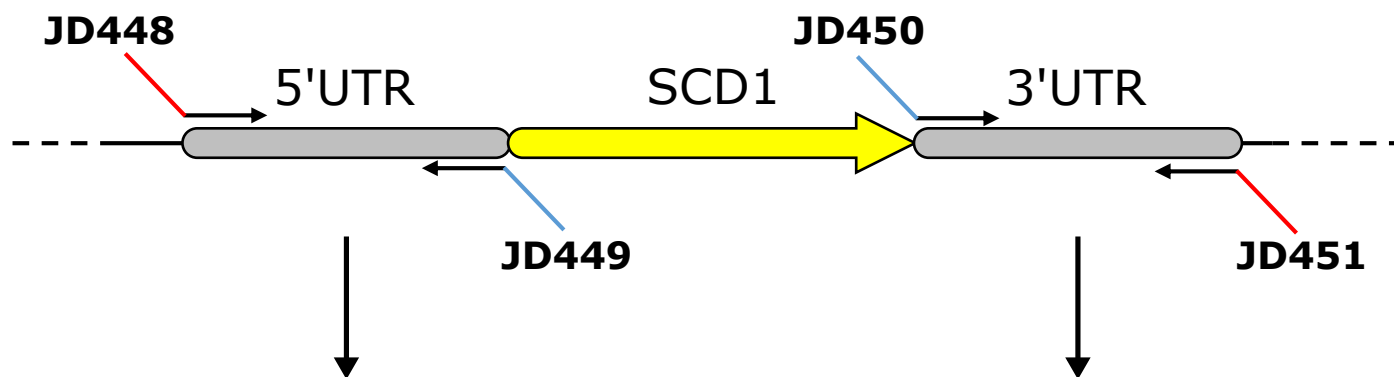

B

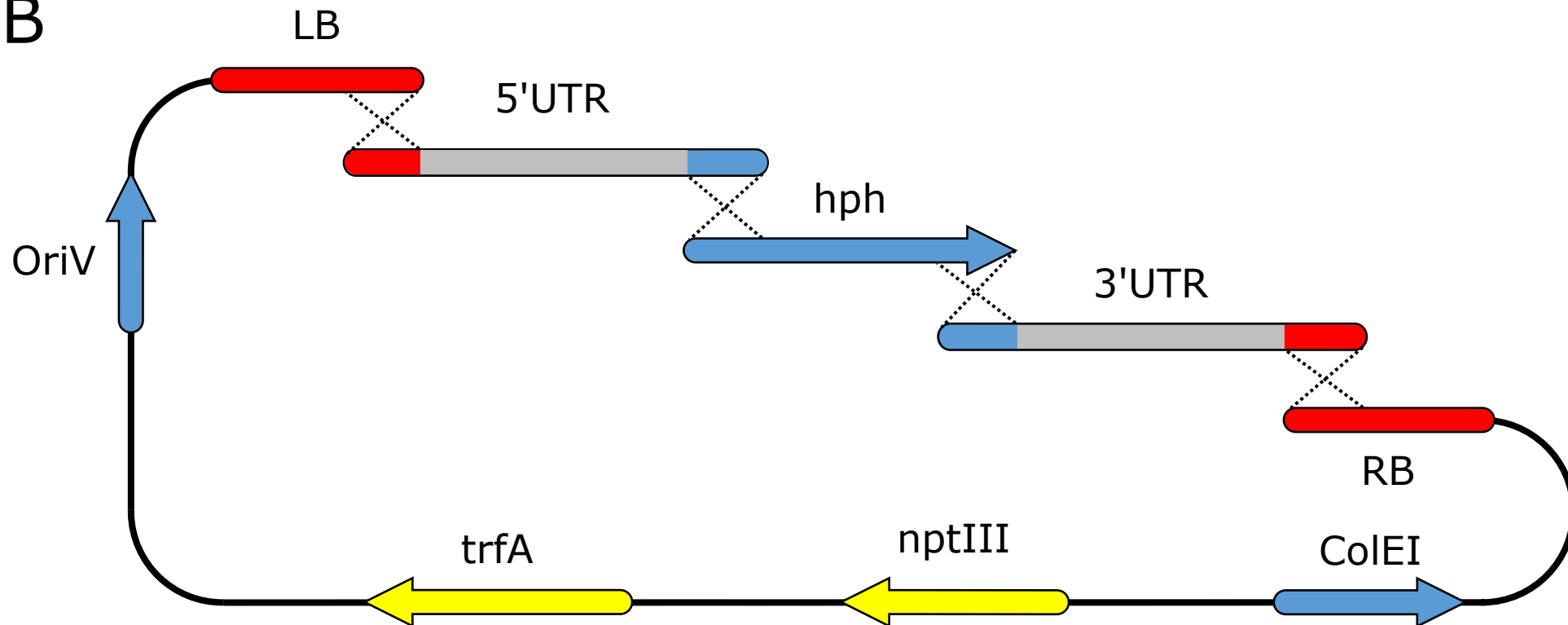

Supplement: Supplementary file 1 [file jof-06-00314-s001.zip › Figure S2.pdf]

736,000 bp

737,000 bp

738,000 bp

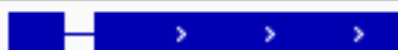

*A/Kewell SCD1*

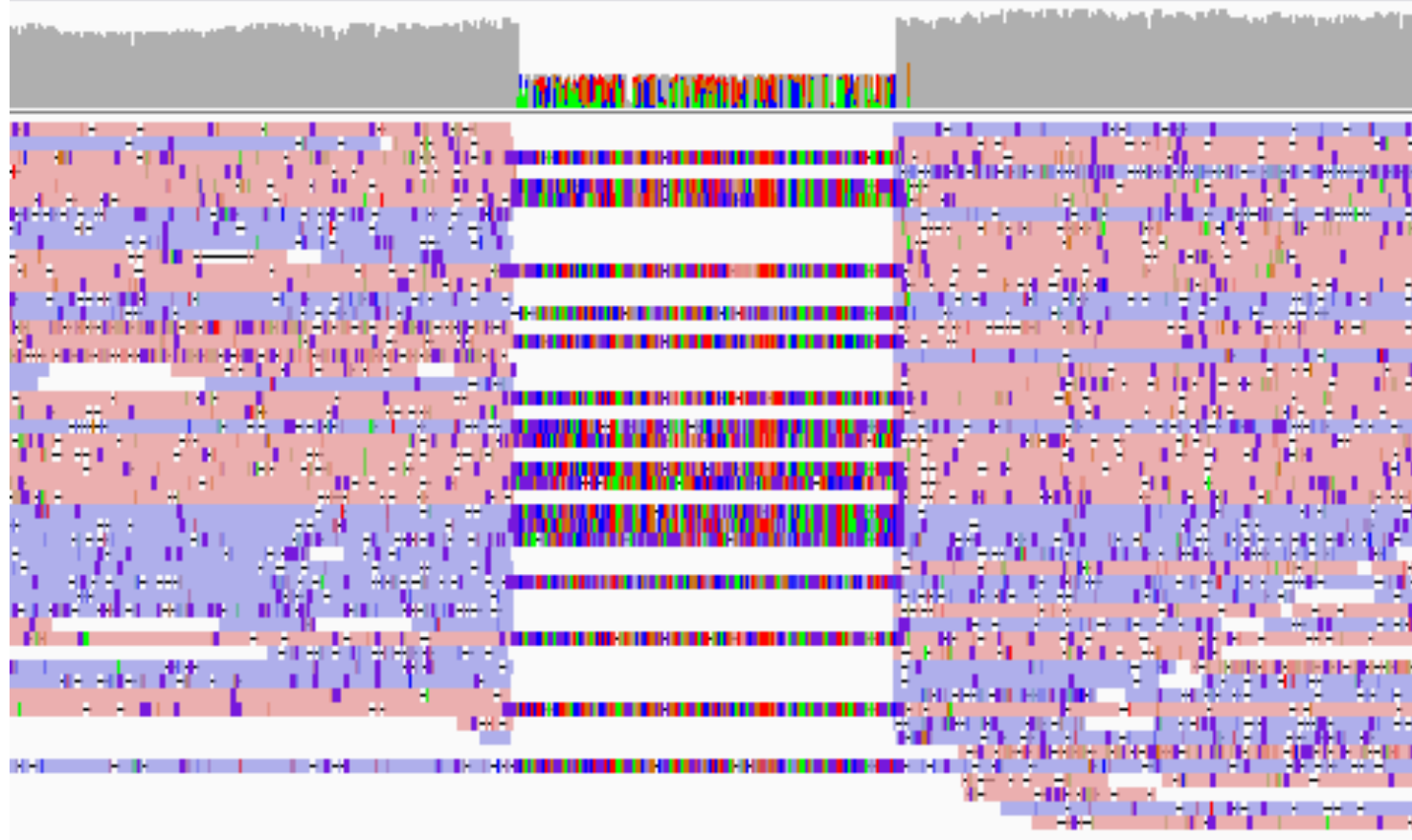

Supplement: Supplementary file 1 [file jof-06-00314-s001.zip › Figure S3.pdf]

736,000 bp

737,000 bp

738,000 bp

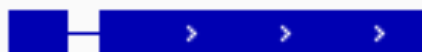

*A/Kewell SCD1*

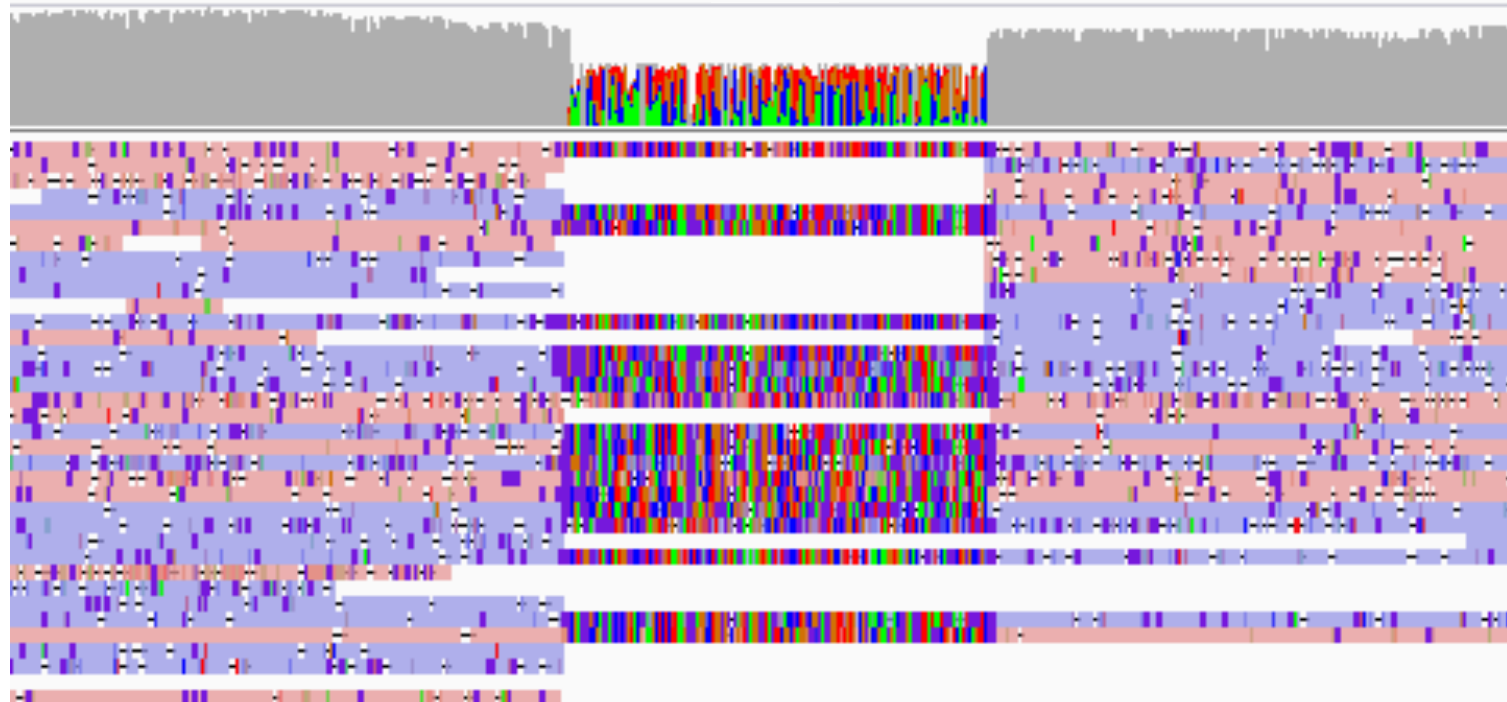

Supplement: Supplementary file 1 [file jof-06-00314-s001.zip › Figure S4.pdf]

**Rep1**

**WT**

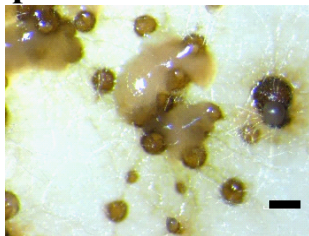

**Ectopic**

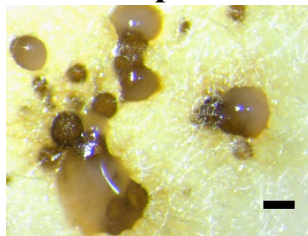

**JD202.9**

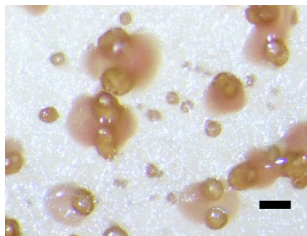

**JD202.22**

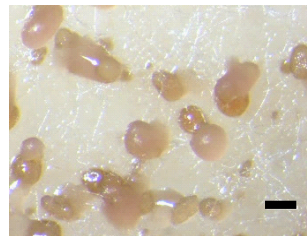

**Rep2**

**WT**

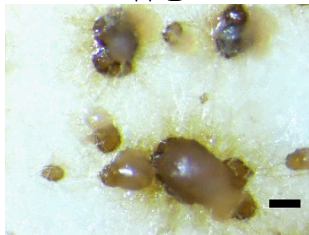

**Ectopic**

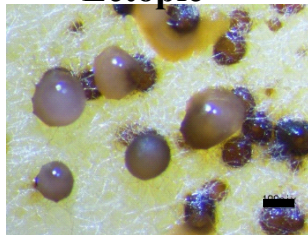

**JD202.9**

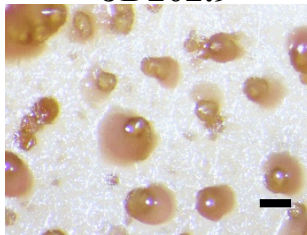

**JD202.22**

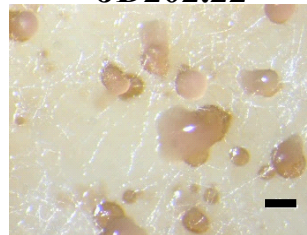

**Rep3**

**WT**

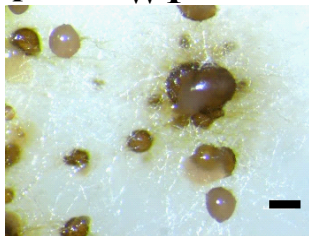

**Ectopic**

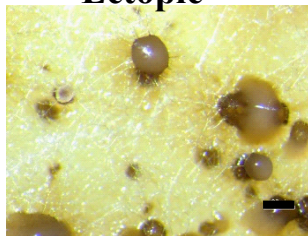

**JD202.9**

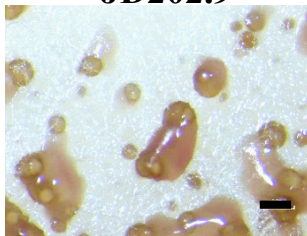

**JD202.22**

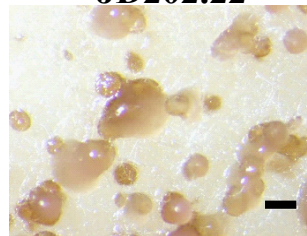

Supplement: Supplementary file 1 [file jof-06-00314-s001.zip › Figure S5.pdf]

Figure S5A

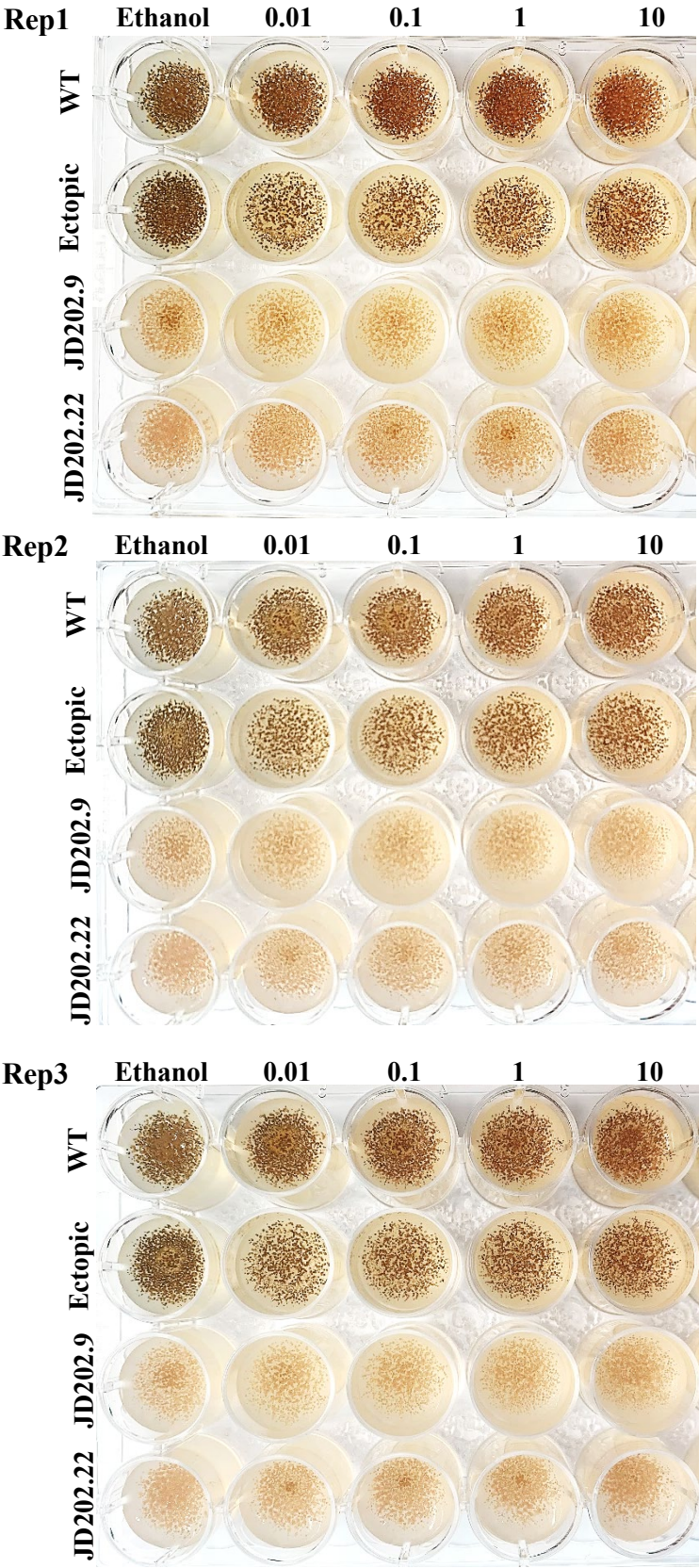

Figure S5B

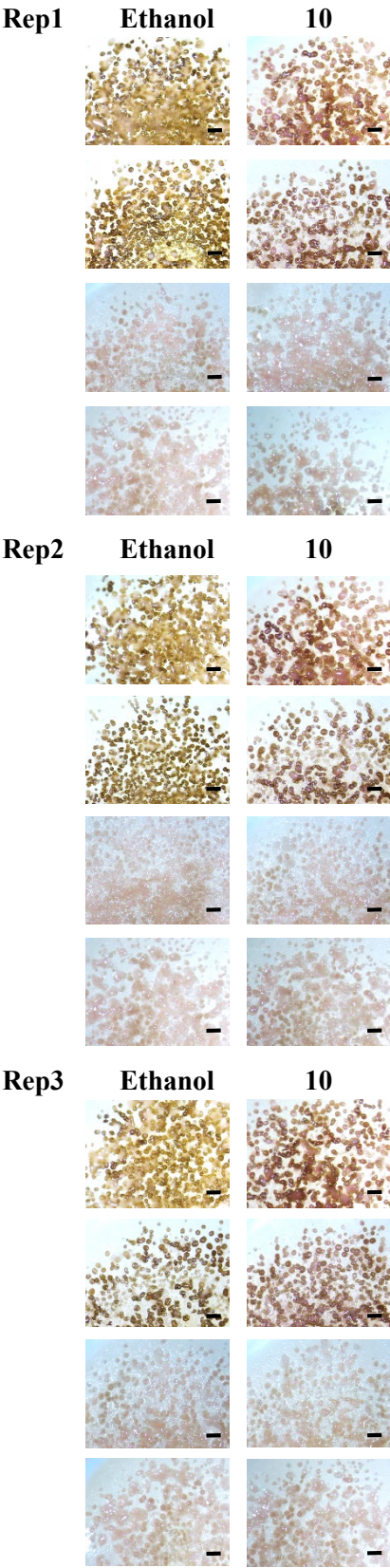

Supplement: Supplementary file 1 [file jof-06-00314-s001.zip › Figure S6.pdf]

**Rep1**

**WT**

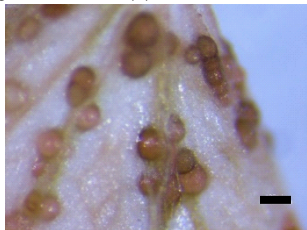

**Ectopic**

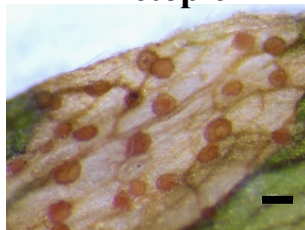

**JD202.9**

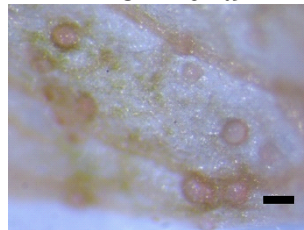

**JD202.22**

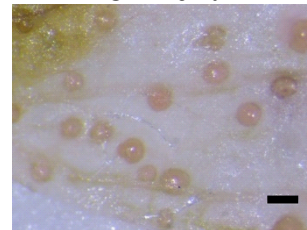

**Rep2**

**WT**

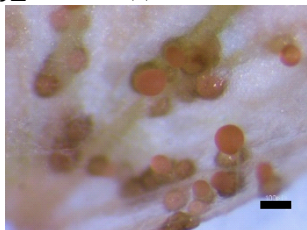

**Ectopic**

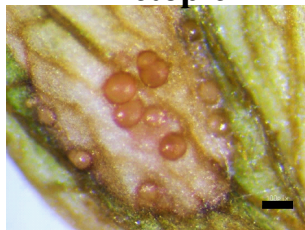

**JD202.9**

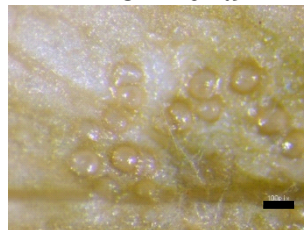

**JD202.22**

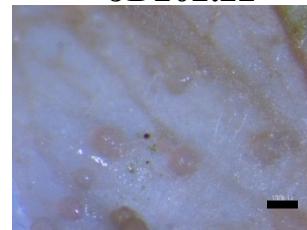

**Rep3**

**WT**

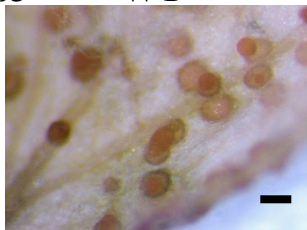

**Ectopic**

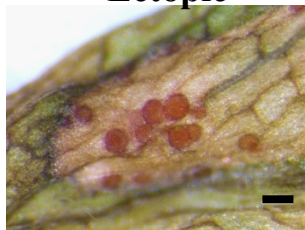

**JD202.9**

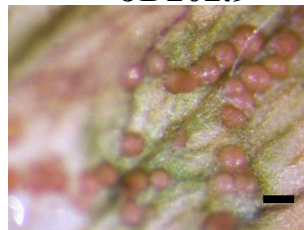

**JD202.22**

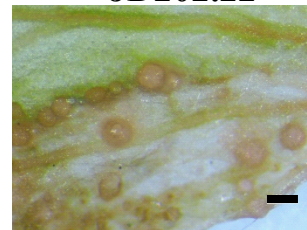

Supplement: Supplementary file 1 [file jof-06-00314-s001.zip › Figure S7.pdf]
